# UBQLN1 mediates sorafenib resistance through regulating mitochondrial biogenesis and ROS homeostasis by targeting PGC1β in hepatocellular carcinoma

**DOI:** 10.1038/s41392-021-00594-4

**Published:** 2021-05-18

**Authors:** Junjie Xu, Lin Ji, Yeling Ruan, Zhe Wan, Zhongjie Lin, Shunjie Xia, Liye Tao, Junhao Zheng, Liuxin Cai, Yifan Wang, Xiao Liang, Xiujun Cai

**Affiliations:** 1 Key Laboratory of Laparoscopic Technology of Zhejiang Province, Department of General Surgery, Sir Run-Run Shaw Hospital, Zhejiang University School of Medicine, Hangzhou, China; 2 Zhejiang Minimal Invasive Diagnosis and Treatment Technology Research Center of Severe Hepatobiliary Disease, Zhejiang Research and Development Engineering Laboratory of Minimally Invasive Technology and Equipment, Hangzhou, China; 3 Zhejiang University Cancer Center, Hangzhou, China; 4 Liangzhu Laboratory, Zhejiang University Medical Center, Hangzhou, China

**Keywords:** Drug development, Gastrointestinal cancer

## Abstract

The treatment for hepatocellular carcinoma (HCC) is promising in recent years, but still facing critical challenges. The first targeted therapy, sorafenib, prolonged the overall survival by months. However, resistance often occurs, largely limits its efficacy. Sorafenib was found to target the electron transport chain complexes, which results in the generation of reactive oxygen species (ROS). To maintain sorafenib resistance and further facilitate tumor progression, cancer cells develop strategies to overcome excessive ROS production and obtain resistance to oxidative stress-induced cell death. In the present study, we investigated the roles of ROS in sorafenib resistance, and found suppressed ROS levels and reductive redox states in sorafenib-resistant HCC cells. Mitochondria in sorafenib-resistant cells maintained greater functional and morphological integrity under the treatment of sorafenib. However, cellular oxygen consumption rate and mitochondria DNA content analyses revealed fewer numbers of mitochondria in sorafenib-resistant cells. Further investigation attributed this finding to decreased mitochondrial biogenesis, likely caused by the accelerated degradation of peroxisome proliferator-activated receptor γ coactivator 1β (PGC1β). Mechanistic dissection showed that upregulated UBQLN1 induced PGC1β degradation in a ubiquitination-independent manner to attenuate mitochondrial biogenesis and ROS production in sorafenib-resistant cells under sorafenib treatment. Furthermore, clinical investigations further indicated that the patients with higher UBQLN1 levels experienced worse recurrence-free survival. In conclusion, we propose a novel mechanism involving mitochondrial biogenesis and ROS homeostasis in sorafenib resistance, which may offer new therapeutic targets and strategies for HCC patients.

## Introduction

Liver cancer is the fourth leading cause of cancer-related death globally,^1^ and with a 5-year survival rate of 18%, liver cancer is the second most lethal tumor.^2^ Among all liver cancer cases, hepatocellular carcinoma (HCC) is the major type and accounts for ~89%. Hepatitis and alcohol abuse were previously considered the leading risk factors for HCC, but at present, nonalcoholic fatty liver disease (NAFLD) is considered an increasing risk factor.^3,4^ NAFLD represents a spectrum of liver pathologies that are strongly associated with metabolic disorder.^5^ Metabolic dysregulation also contributes to the progression from NAFLD to HCC.^6^ Because reactive oxygen species (ROS) is intimately associated with metabolic processes, ROS likely plays a significant role in HCC development, particularly those HCC progressed from NAFLD.^7^

Sorafenib, the first FDA-approved multi-target tyrosine kinase inhibitor (TKI) that inhibits the tumor angiogenesis and demonstrates clear clinical benefit for advanced HCC patients. The efficacy of sorafenib was confirmed in two large clinical trials and shown to be superior to dozens of other molecular agents.^8,9^ Despite its efficacy, the clinical effectiveness of sorafenib has been largely limited by the development of resistance.^10^ The mechanisms of resistance include metabolic reprogramming, epithelial–mesenchymal transition,^11^ dysregulation of PI3K/AKT and JAK/STAT pathways,^12,13^ and hypoxia-inducible response as a result of inhibition of angiogenesis by sorafenib.^14–16^ Hypoxia is a well-known cancer feature closely related to ROS generation that promotes malignant progression and other complex biological consequences.^17^ Importantly, sorafenib was reported to directly target electron transport chain (ETC) complexes, which resulted in ROS generation.^18,19^ However, the roles of ROS in sorafenib efficacy and resistance remain largely elusive and need to be clarified.

Mitochondria produce the majority of cellular ROS, particularly in response to hypoxia.^20^ Recently, mitochondrial biogenesis was shown to be tightly linked to ROS production.^21^ Studies found that the upregulation of mitochondrial content resulted in higher ROS levels under stress.^22,23^ Whether mitochondrial biogenesis-mediated ROS levels play a role in sorafenib resistance in HCC remains to be determined.

In this study, we found alleviated ROS responses in sorafenib-resistant HCC cells due to decreased mitochondrial biogenesis. Mechanistic studies revealed that upregulation of UBQLN1 in sorafenib-resistant HCC cells expedited the proteasome-mediated protein degradation of peroxisome proliferator-activated receptor γ coactivator 1β (PGC1β), contributing to decreased mitochondrial biogenesis and ROS generation, finally inducing sorafenib resistance. These findings may provide a novel mechanism involving mitochondrial biogenesis and ROS homeostasis in sorafenib resistance, which may offer new therapeutic targets and strategies for HCC patients.

## Results

### Decreased ROS levels in sorafenib-resistant HCC cells

**Fig. 1:**
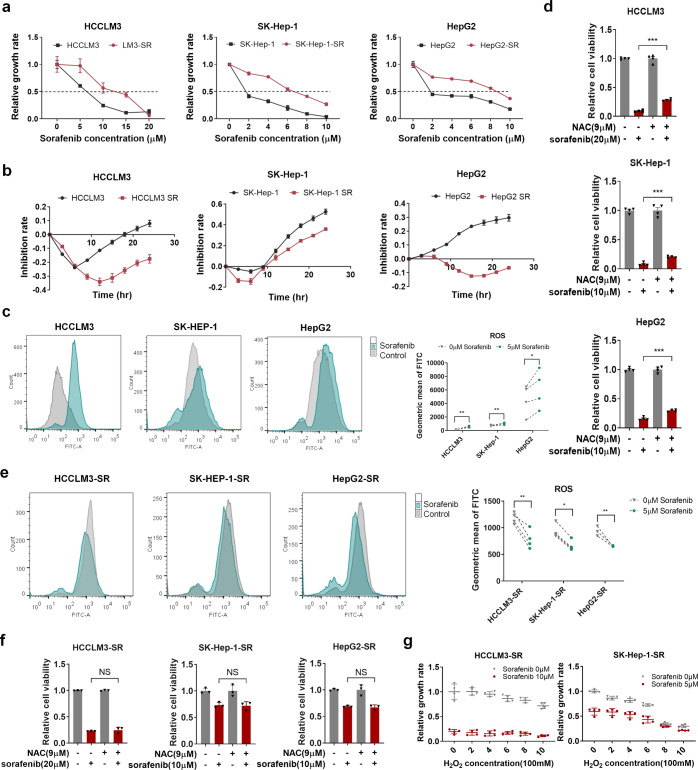
Decreased ROS levels in sorafenib-resistant HCC cells. **a** Cells were treated with different concentrations of sorafenib for 48 h. Cell viability was measured using the CCK-8 assay. Resistant cells had higher IC50s than their parental counterparts. **b** Real-time cell analysis revealed the inhibition rates of sorafenib (HCCLM3: 10 µM, SK-Hep-1: 5 µM, HepG2: 5 µM) over time. **c**, **e** Intracellular ROS levels were measured using a DCFH-DA probe via flow cytometry with or without 48 h of sorafenib treatment (5 µM) in parental and resistant cells. **d**, **f** Cell viability was measured by CCK-8 assay with or without sorafenib treatment and ROS scavenger *N*-acetyl-cysteine (NAC), which was added to the media 3 h before sorafenib treatment. **g** Cell viability was measured by CCK-8 assay with or without sorafenib treatment and hydrogen peroxide (H_2_O_2_). *p < 0.05, **p < 0.01, and ***p < 0.001. NS not statistically significant

To mimic the biological process of sorafenib resistance in HCC patients, an in vitro model was established in this study, as described previously.^24,25^ Briefly, resistance was established in three HCC cell lines via exposure to gradually increasing sorafenib concentrations in the media for ~6 months. Three pairs of parental and resistant cell lines were established, with different levels of tolerance to sorafenib (Fig. 1a, b).

Sorafenib targets multiple tyrosine kinases to retard tumor angiogenesis, inducing intratumoral hypoxia and ROS generation, which are closely related to each other and may play pivotal roles in developing drug resistance.^15^ Thus, we evaluated the effects of sorafenib on ROS levels in our in vitro model and found that sorafenib treatment induced ROS generation in parental cells (Fig. 1c), consistent with prior reports.^18,19^ Notably, the ROS scavenger *N*-acetyl-cysteine (NAC)^26^ partially rescued the cell death induced by sorafenib in parental HCC cells (Fig. 1d), suggesting that sorafenib might induce cell death partially through increasing ROS levels. Intriguingly, sorafenib exerted the opposite effect and decreased ROS levels in resistant HCC cells (Fig. 1e). Importantly, NAC could not rescue the cell death induced by sorafenib in resistant HCC cells. However, increasing concentrations of hydrogen peroxide (a ROS inducer) could partially rescue the resistance to sorafenib (Fig. 1f, g).

Thus, the decreased ROS levels in response to sorafenib treatment observed in resistant cells might be critical for the development of sorafenib resistance in HCC.

### Sorafenib-resistant cells retain better mitochondrial function and integrity with less mitochondrial content and respiratory capacity

**Fig. 2:**
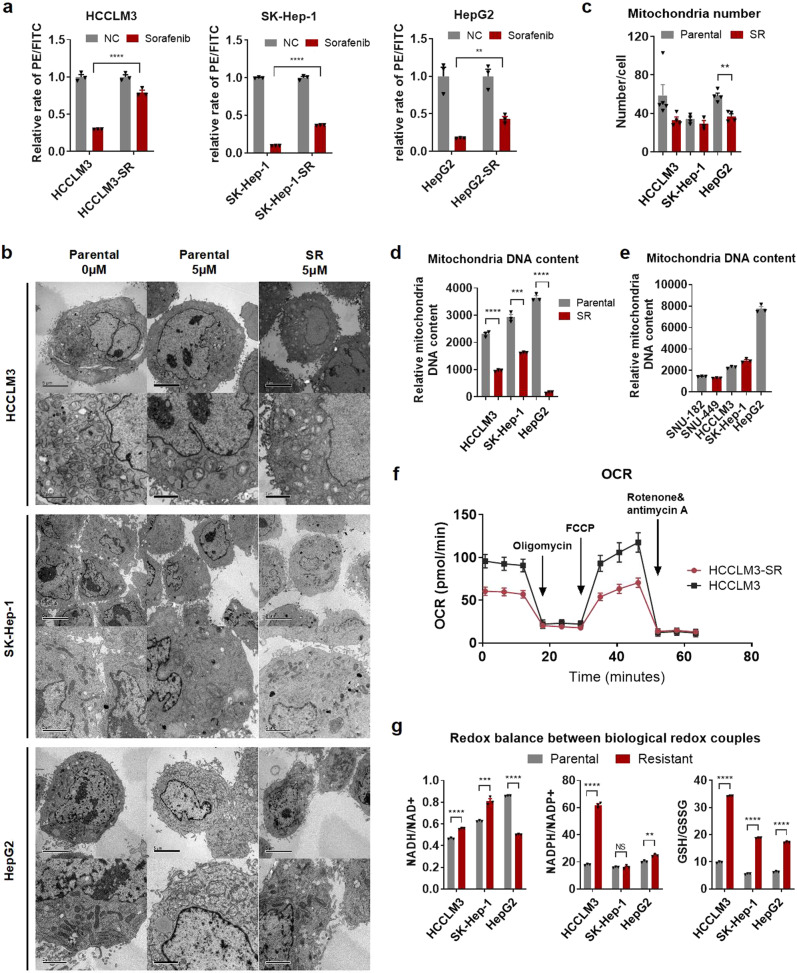
Sorafenib-resistant cells retains better mitochondrial function and integrity with less mitochondrial content and respiratory capacity. **a** Mitochondrial membrane potential (MMP) was measured using a Mitochondria Staining Kit (JC-1). Flow cytometry revealed decreased mitochondrial membrane potentials (MMP) after 48 h of sorafenib treatment (5 µM) in parental cells, and much less in resistant cells. **b** Representative transmission electron microscopy (TEM) images of mitochondrial morphological changes during sorafenib treatment. Mitochondria in resistant cells maintained an intact morphology during sorafenib treatment, whereas those in parental cells were altered. Scale bar, 5 µm. **c** The numbers of mitochondria were determined via TEM. **d**, **e** Mitochondrial DNA content was measured by qRT-PCR. **f** Oxygen consumption rate (OCR) analysis using Seahorse analysis revealed compromised mitochondrial function in HCCLM3 cells. **g** Redox balances between biological redox couples of GSH/GSSG, NADPH/NADP, and NAD/NADH in parental and sorafenib-resistant cells were monitored. **p < 0.01, ***p < 0.001, and ****p < 0.0001. NS not statistically significant

Previous studies found that sorafenib targets mitochondrial ETC complexes to induce ROS.^18,19^ To delineate the underlying mechanisms of the discrepancies in ROS in response to sorafenib, we focused on the role of mitochondria in the process of sorafenib resistance because mitochondria are the main source of ROS in cells.^27^ We first measured the mitochondrial membrane potential (MMP) via flow cytometry and found that the MMP was decreased in response to sorafenib treatment in both cell lines, but to a significantly lesser extent in resistant cells (Fig. 2a). Morphological analysis of mitochondria with transmission electron microscopy (TEM) showed that mitochondrial cristae in parental cells were partly disintegrated after 48 h of sorafenib treatment, while mitochondria largely remained intact in resistant cells (Fig. 2b), indicating better mitochondrial function under the stress of sorafenib treatment in resistant cells. Interestingly, the number of mitochondria and mitochondrial DNA (mtDNA) levels were lower in resistant cells than in parental cells (Fig. 2c, d). We further measured mtDNA levels in two other HCC cell lines, SNU182 and SNU449, which were reported primarily resistant to sorafenib,^28^ and found that mtDNA levels were much lower in SNU182 and SNU449 cells compared with those in the three parental HCC cell lines (Fig. 2e). These results suggest that lower mitochondrial content in resistant cells might result in less ROS production when treated with sorafenib. Moreover, the oxygen consumption rate (OCR) was lower in resistant cells than in parental cells (Fig. 2f), indicating a reduced mitochondrial respiratory capacity, likely as a result of the reduced mitochondrial number in resistant cells.

The antioxidant system in most drug-resistant cancer cells is complicated.^29^ Sorafenib, as a multi-kinase inhibitor, may regulate redox homeostasis in drug-resistant cancer cells. Thus, we monitored the redox balance between biological redox couples, such as GSH/GSSG, NADPH/NADP, and NAD/NADH in parental and resistant cells. In general, compared with parental cell lines, a relative reductive state was found in resistant cell lines (Fig. 2g). Interestingly, NADH/NAD+ in HepG2 cells and NADPH/NADP+ in SK-Hep-1 cells showed inconsistent trends, indicating a general reductive but cell line-specific redox homeostasis in sorafenib resistance.

Taken together, these results indicate that resistant cells retain better mitochondrial function and integrity with less mitochondrial content and respiratory capacity, resulting in reduced ROS production in response to sorafenib treatment and sustained sorafenib resistance.

### Accelerated PGC1β degradation inhibits mitochondrial biogenesis and ROS generation in sorafenib-resistant cells

**Fig. 3:**
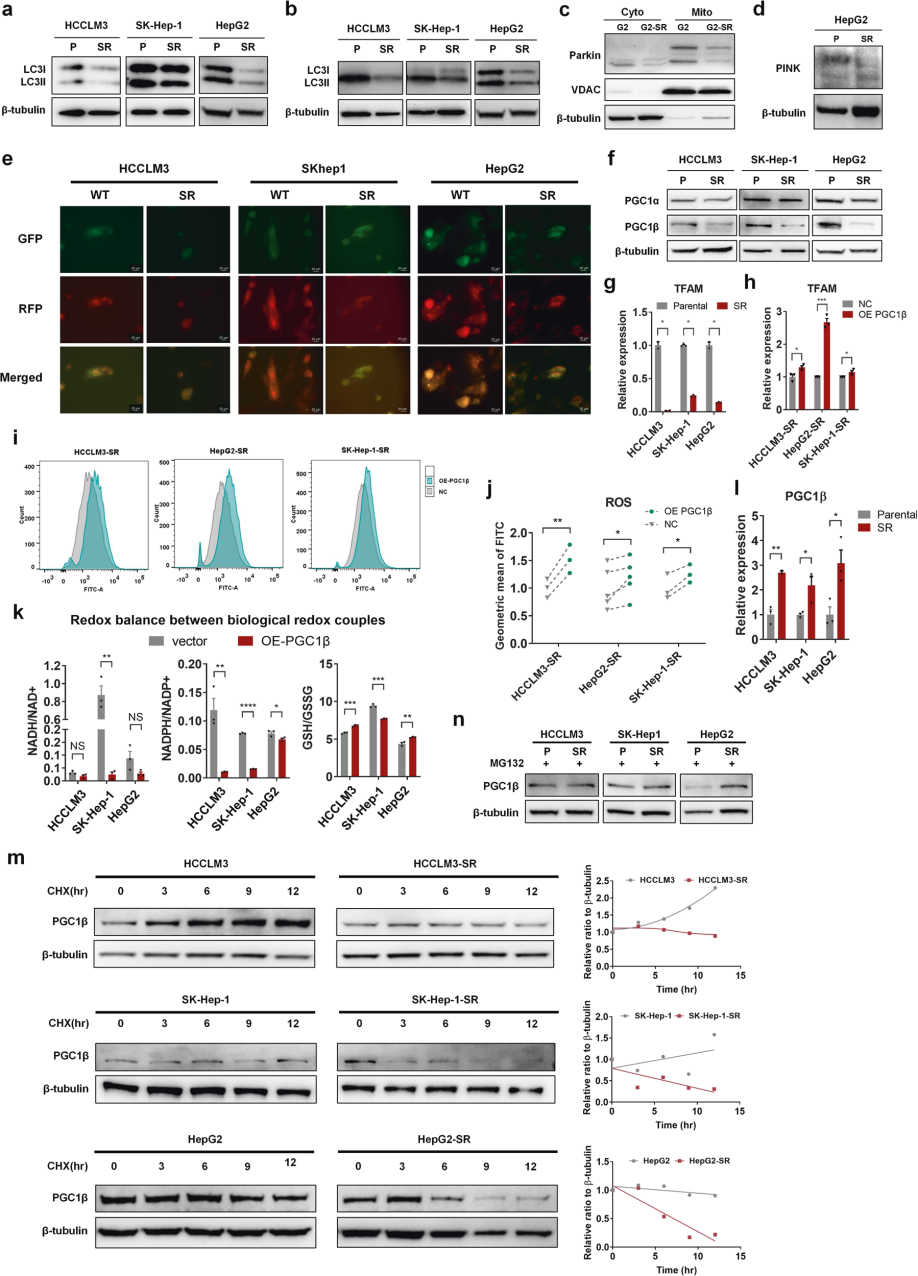
Accelerated peroxisome proliferator-activated receptor γ coactivator 1β (PGC1β) degradation inhibits mitochondrial biogenesis and ROS generation in sorafenib-resistant cells. **a** WB analysis of LC3I and LC3II in parental and resistant cells with sorafenib treatment. **b** WB analysis of LC3I and LC3II in parental and resistant cells incubated with chloroquine (20 µM) 3 h prior to sorafenib treatment. **c** Parental and resistant HepG2 cells were treated with sorafenib. Mitochondria were fractionated and subjected to WB. WB analysis of Parkin, and VDAC showed higher levels of Parkin translocated into mitochondria in parental than resistant HepG2 cells. **d** WB analysis of PINK in parental and resistant HepG2 cells treated with sorafenib. **e** Cells were transfected with LC3-GFP-RFP to demonstrate autophagic flux. Fluorescence microscopy revealed the presence of more autophagic vacuoles in parental than resistant cells. **f** WB analysis of PGC1α and PGC1β in parental and resistant HepG2 cells with sorafenib treatment. **g**, **h** qRT-PCR assays determining the mRNA levels of mitochondrial transcription factor A (TFAM). **i**, **j** ROS levels were measured using a DCFH-DA probe via flow cytometry with or without PGC1β overexpression in resistant cells treated with sorafenib. **k** Redox balances between biological redox couples of GSH/GSSG, NADPH/NADP, and NAD/NADH were monitored in HCC cells with or without PGC1β overexpression. **l** qRT-PCR assays determining the mRNA levels of PGC1β in parental and resistant cells treated with sorafenib. **m** WB analysis of PGC1β in parental and resistant cells treated with sorafenib and CHX (100 µg/ml). **n** WB analysis analysis of PGC1β in parental and resistant cells treated with sorafenib and MG132(20 µM) for 3 h before being harvested. *p < 0.05, **p < 0.01, ***p < 0.001, and ****p < 0.0001. NS not statistically significant

Cells have developed finely tuned and complex mechanisms to adapt to stress conditions and metabolic demand alterations by modulating mitochondrial number and function.^30^ Mitochondria and cellular homeostasis are accurately coordinated by mitochondrial biogenesis and clearance. To elucidate the underlying mechanisms of the reduced mitochondrial content in resistant cells, we first evaluated mitochondria degradation. Mitophagy is a principle mechanism for mitochondria degradation and reduction of ROS stress.^31^ We measured mitophagy levels in parental and resistant cells, and found that LC3II (reflects autophagic activity) levels were lower in resistant cells under sorafenib treatment, with or without chloroquine (a classic inhibitor of autophagy that alters the acidic environment of lysosomes thus blocks the binding of autophagosomes to lysosomes; Fig. 3a, b). Moreover, levels of the mitophagy-specific markers PARKIN and PINK were also reduced in resistant cells (Fig. 3c, d). Fluorescence imaging further revealed a lower autophagic flux in resistant cells than parental cells under sorafenib treatment (Fig. 3e). These results indicated that mitophagy was decreased in resistant cells under sorafenib treatment, which is unlikely to explain the lower mitochondrial content in resistant cells.

We next examined mitochondrial biogenesis in the pairs of HCC cell lines. The PGC1 family of transcriptional coactivators act as master regulators of mitochondrial biogenesis, with PGC1α and PGC1β regulating mitochondrial transcription factor A (TFAM) to maintain mtDNA levels and mitochondrial function.^32^ Thus, we first evaluated PGC1α and PGC1β protein levels, and found that PGC1β expression was reduced in the resistant cell lines, while little change was observed with PGC1α expression (Fig. 3f). Furthermore, the mRNA levels of TFAM were also decreased in resistant cells compared with those in parental cells (Fig. 3g), and overexpressing PGC1β in resistant cells increased TFAM mRNA levels (Fig. 3h) and ROS production in response to sorafenib (Fig. 3i, j). Moreover, overexpressing PGC1β in resistant cells resulted in a general oxidative, but cell line-specific redox homeostasis in response to sorafenib (Fig. 3k).

Together, these results suggest that the decreased PGC1β in resistant cells might result in decreased mitochondrial biogenesis and ROS generation in response to sorafenib treatment.

We then investigated the upstream regulation of PGC1β in sorafenib resistance in HCC. While PGC1β protein levels were decreased in resistant cells, the mRNA levels of PGC1β were increased compared with those in parental cells (Fig. 3l), suggesting a posttranscriptional regulation of PGC1β in sorafenib resistance. Moreover, analyses of PGC1β protein stability using cycloheximide to inhibit de novo protein synthesis revealed an accelerated PGC1β degradation in resistant cells (Fig. 3m). Furthermore, blocking the proteasome with the addition of MG132 increased PGC1β protein in resistant cells to equivalent levels or higher levels in parental cells (Fig. 3n). These results suggest that proteasome-mediated PGC1β degradation is increased in resistant cells compared with that in parental cells.

Taken together, these findings indicated that PGC1β degradation was increased in resistant cells, leading to decreased mitochondrial biogenesis and ROS generation in response to sorafenib treatment.

### UBQLN1 regulates PGC1β degradation in sorafenib resistance

**Fig. 4:**
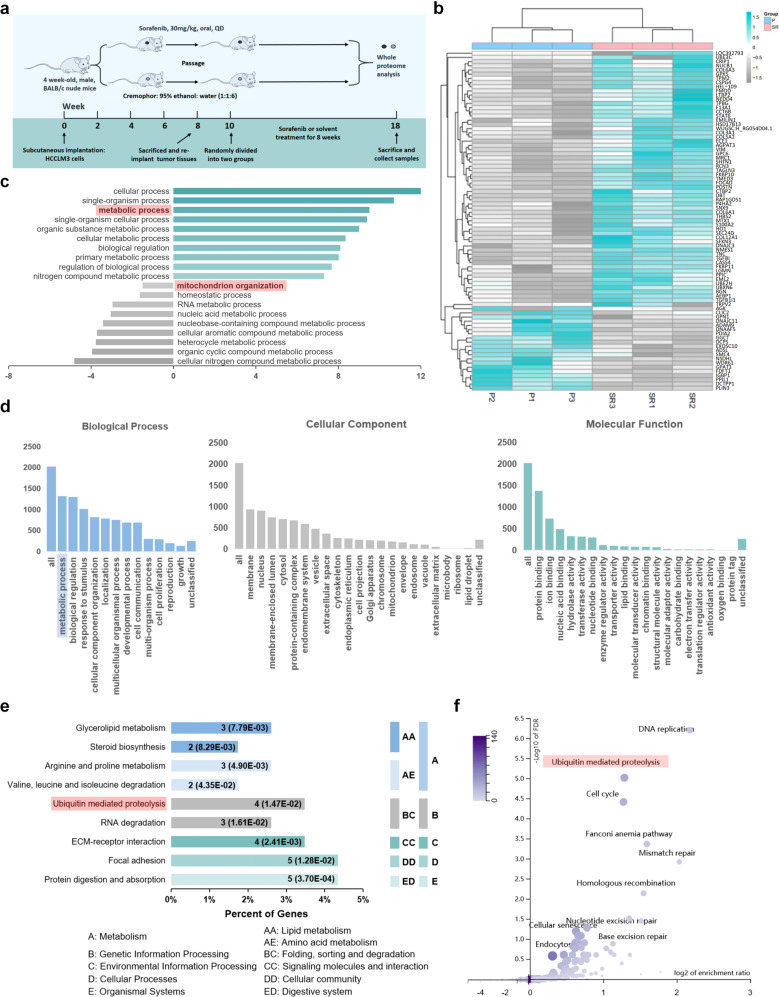
The role of metabolic process and ubiquitin-mediated proteolysis in sorafenib resistance. **a** Flow chart of the establishment of a orthotopic sorafenib-resistant mouse model. **b** The heatmap shows 80 proteins identified via mass spectrum analysis, with differential expressions (*p* < 0.05) between tumors harvested from the parental and sorafenib-resistant models described in **a**. P parental, SR sorafenib-resistant. **c** GO analysis of the deregulated proteins shown in **b** indicated that metabolic process and mitochondrion organization were changed in resistant cells (*p* = 0.0105). **d** GO analysis using GSE94550 from GEO datasets. Biological process, cellular component, and molecular function category were represented. **e** KEGG pathway analysis of the deregulated proteins shown in **b** indicated that the ubiquitin–proteasome pathway was altered in sorafenib-resistant cells (*p* = 0.0147). **f** KEGG pathway analysis using GSE94550 from GEO datasets demonstrated that ubiquitin-mediated proteolysis was involved in resistance development (*p* = 5.9138e−8)

To dissect the mechanisms underlying sorafenib resistance in vivo and further elucidate the detailed mechanisms of accelerated PGC1β degradation in sorafenib resistance in HCC, we constructed a subcutaneous sorafenib-resistant HCC mouse model using HCCLM3 cells (Fig. 4a), according to previous studies.^12,25,33^ Whole-proteome analysis by mass spectrometry found 80 differentially expressed proteins between parental and resistant tumors (*p* < 0.05; Fig. 4b). Subsequent GO analysis of tumors indicated that altered metabolic process and mitochondrion organization were involved in sorafenib resistance (Fig. 4c), consistent with in vitro findings. Importantly, GO analysis of the public dataset GSE94550 also indicated that metabolic process was significantly altered during the development of sorafenib resistance (Fig. 4d). Notably, KEGG pathway analysis further suggested that ubiquitin-mediated proteolysis might play roles in sorafenib resistance (Fig. 4e), which was also identified in the KEGG pathway analysis of the GSE94550 dataset (Fig. 4f).^34^

**Fig. 5:**
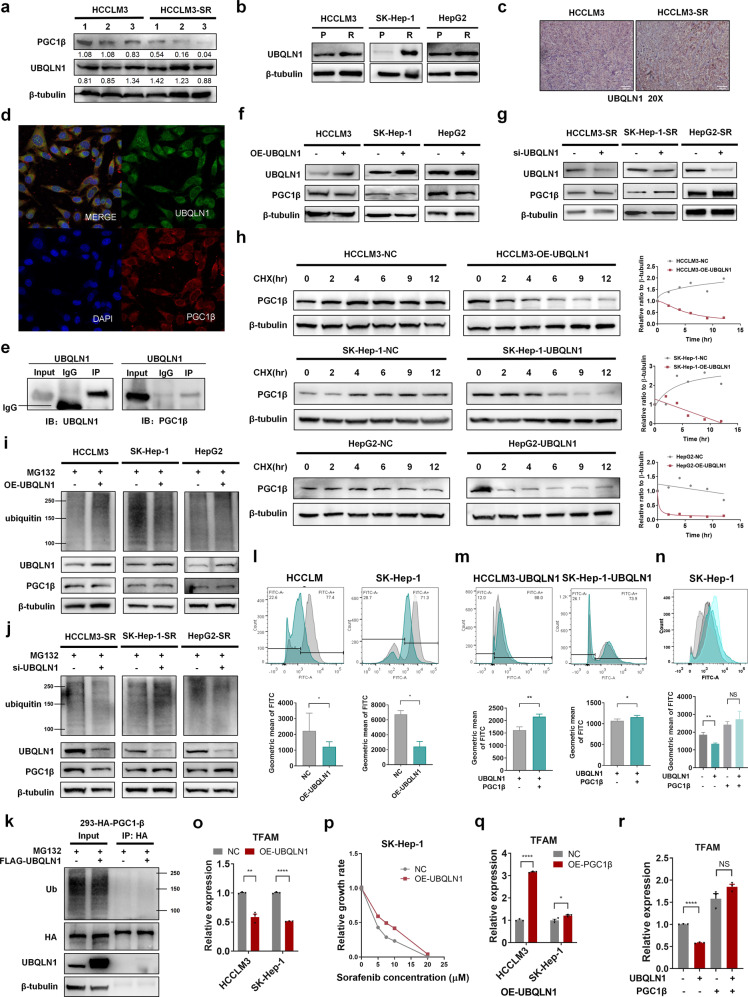
The role of UBQLN1 regulated PGC1β degradation in sorafenib resistance. **a** WB analysis of PGC1β and UBQLN1 in tumors harvested from the parental and sorafenib-resistant models described in Fig. 4a. **b** WB analysis of UBQLN1 in parental and resistant cells with sorafenib treatment. **c** Representative immunohistochemistry (IHC) images of UBQLN1 in tumors harvested from the parental and sorafenib-resistant models described in Fig. 4a. **d** Fluorescence confocal microscopy revealed the colocalization of UBQLN1 and PGC1β. **e** WB analysis of PGC1β immunoprecipitated by UBQLN1 in resistant HepG2 cells. **f** WB analysis of PGC1β and UBQLN1 in parental cells with or without UBQLN1 overexpression. **g** WB analysis of PGC1β and UBQLN1 in resistant cells with or without UBQLN1 silencing. **h** WB analysis of PGC1β in cells overexpressed with vector or UBQLN1 and treated with sorafenib and CHX (100 µg/ml). **i** WB analysis of ubiquitin, PGC1β, UBQLN1, and β-tubulin in parental cells with or without UBQLN1 overexpression. Cells were treated with MG132 (20 µM) for 3 h before being harvested. **j** WB analysis of ubiquitin, PGC1β, and UBQLN1 in resistant cells with or without UBQLN1 silencing. Cells were treated with MG132(20 µM) for 3 h before being harvested. **k** Immunoprecipitation and WB analysis of MYC, PGC1β, and UBQLN1 in 293T-OE-MYC-ub cells overexpressed with or without UBQLN1 overexpression. Cells were treated with MG132 (20 µM) for 3 h before being harvested. **l**–**n** ROS levels were measured using a DCFH-DA probe via flow cytometry with or without UBQLN1 or PGC1β overexpression in parental cells treated with sorafenib. **o**, **q**, **r** qRT-PCR assays determining the mRNA levels of mitochondrial transcription factor A (TFAM) with or without UBQLN1 or PGC1β overexpression in parental cells treated with sorafenib. **p** Cell viability was measured using the CCK-8 assay in parental SK-Hep-1 cells treated with sorafenib. *p < 0.05, **p < 0.01, ***p < 0.0001, and ****p < 0.0001. NS not statistically significant

As PGC1β stability was decreased in vitro in sorafenib resistance in a proteasome-mediated process, this process is likely regulated by ubiquitin-mediated proteolysis as with most cellular proteins. We found that PGC1β expression was significantly decreased in sorafenib-resistant tumors in vivo (Fig. 5a), consistent with the in vitro results.

Protein ubiquitination and degradation is regulated by a series of enzymes: ubiquitin activating enzymes E1, E2 and the substrate targeting ubiquitin ligase E3. Ubiquitin E3 ligases are involved in multiple biological processes, including drug resistance by catalyzing protein ubiquitination and promoting protein degradation.^35^ Protein ubiquitination can also be regulated by poly-ubiquitin chain chaperones, such as ubiquilins.^36^ Our prior research indicated that UBQLN1 was increased in sorafenib-resistant cells (data unpublished). We also detected elevated UBQLN1 levels in both sorafenib-resistant HCC cells in vitro and tumors in vivo (Fig. 5a–c). UBQLN1 has three functional domains: a ubiquitin-like domain (UBL) at the N-terminus, a ubiquitin-associated domain (UBA) at the C-terminus, and STI chaperone-like regions in the central region.^37^ Studies found that UBQLN1 assists in proteasomal degradation by binding to poly-ubiquitin chains on substrate proteins through its UBA domain, and shuttling the substrate to the 19s proteasome via its UBL-S5a cap interaction. UBQLN1 also recruits the ubiquitin E3 ligase to ubiquitinate its bound target. We speculated whether PGC1β may be regulated by UBQLN1.

Confocal microscopy and immunoprecipitation assays demonstrated the colocalization of UBQLN1 and PGC1β, and the direct interaction between them (Fig. 5d, e). Furthermore, overexpression of UBQLN1 led to decreased PGC1β levels (Fig. 5f), while silencing UBQLN1 led to increased PGC1β levels (Fig. 5g). Cycloheximide assay further showed that overexpression of UBQLN1 accelerated PGC1β degradation in parental HCC cells (Fig. 5h). Importantly, the effects of UBQLN1 on PGC1β levels could be eradicated by MG132 treatment (Fig. 5i, j), indicating that UBQLN1 regulates PGC1β by proteasome-mediated degradation.

Of note, the overall ubiquitination levels were increased in HCCLM3 and HepG2 cells, whereas they decreased in SK-Hep-1 cells, following overexpression of UBQLN1 (Fig. 5i). Similarly, silencing UBQLN1 decreased the overall ubiquitination levels in HCCLM3 and HepG2 cells, but increased ubiquitination in SK-Hep-1 cells (Fig. 5j). These results suggested a cell line-specific effect of UBQLN1 on general ubiquitination levels, as UBQLN1 was reported to have dual functions that help to ubiquitinate its bound target and shuttle the substrate to the proteasome. To demonstrate the effect of UBQLN1 on ubiquitination of PGC1β, HA-PGC1β overexpressed 293T cells were prepared for further analysis. As shown in Fig. 5k, the ubiquitination of PGC1β remained unchanged upon overexpression of UBQLN1, indicating that UBQLN1 facilitates the interaction between PGC1β and proteasome in a ubiquitination-independent manner, thus accelerate its the proteolysis of PGC1β.

Overexpression of UBQLN1 resulted in reduced ROS levels (Fig. 5l), which were partially rescued by PGC1β overexpression (Fig. 5m). Rescue assays further demonstrated that overexpression of PGC1β partially rescued UBQLN1-induced ROS reduction (Fig. 5n). Moreover, overexpression of UBQLN1 also reduced TFAM mRNA levels in parental cells treated with sorafenib (Fig. 5o), while it partially protected cells from sorafenib-induced cell death (Fig. 5p). Importantly, PGC1β overexpression partially rescued the decreased TFAM by UBQLN1 overexpression (Fig. 5q, r). These results indicate that UBQLN1 inhibits mitochondrial biogenesis and ROS generation in a PGC1β-dependent manner.

**Fig. 6:**
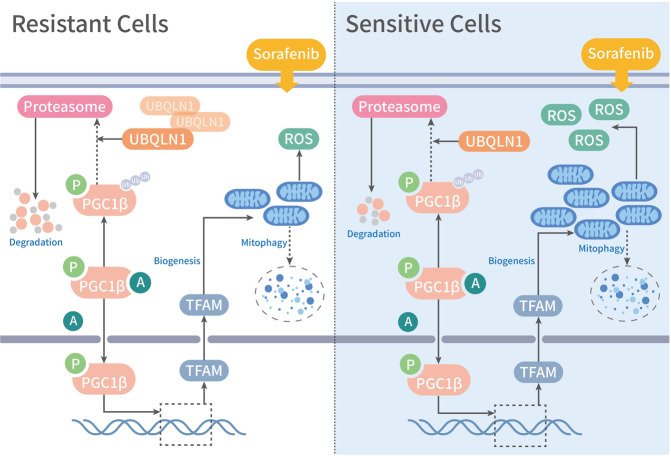
Proposed mechanistic depiction of sorafenib resistance. The alleviated ROS responses in sorafenib-resistant HCC cells was due to decreased mitochondrial biogenesis. Mechanistic dissection indicated that upregulation of UBQLN1 in sorafenib-resistant HCC cells accelerated the protein degradation of PGC1β, contributing to the decreased mitochondrial biogenesis and ROS generation, finally inducing sorafenib resistance

Taken together, these findings strongly support a role of UBQLN1 in mediating PGC1β degradation and subsequent inhibition of mitochondrial biogenesis and ROS generation in sorafenib resistance (Fig. 6).

### The role of UBQLN1 in promoting HCC prognosis and sorafenib resistance in vivo

**Fig. 7:**
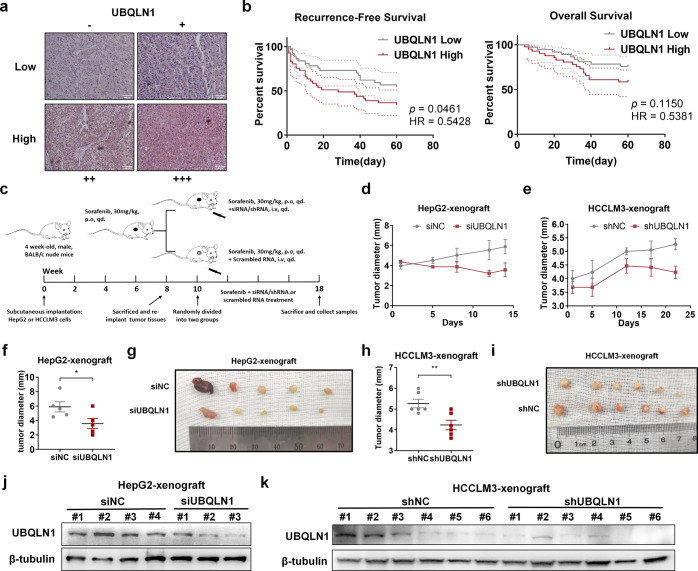
The role of UBQLN1 in HCC prognosis and sorafenib resistance in vivo. **a** Representative IHC images of UBQLN1 expression in HCC clinical samples. Patients were divided into two groups. The low expression group included patients with negative and weak expression of UBQLN1, and the high expression group included patients with moderate and strong expression of UBQLN1. **b** The survival curves of recurrence-free survival and overall survival were calculated using the log-rank test. **c** Flow chart of the establishment of sorafenib-resistant mouse models. siRNA transfection was adopted to reduce UBQLN1 levels in tumors. **d**, **e** The size of xenograft was measured in vivo every 3–4 days using Vernier caliper. **f** The size of HepG2-xenograft was measured in vivo by caliper prior to sacrifice. **g** Subcutaneous HepG2-xenograft from both groups were harvested. **h** The size of HCCLM3-xenograft was measured in vivo by Vernier caliper prior to sacrifice. **i** Subcutaneous HCCLM3-xenograft from both groups were harvested. **j**, **k** Western blot analysis of UBQLN1 and β-tubulin in tumors harvested from the mouse models described in **c**. *p < 0.05 and **p < 0.01. NS not statistically significant. UBQLN1: UBQLN1

To validate the role of UBQLN1 in clinical HCC patients, 78 patients with HCC with complete prognosis information were enrolled; the clinical features of these patients are presented in Table 1. Immunohistochemistry was performed to evaluate UBQLN1 levels in tumor tissues, and patients were divided into two groups according to UBQLN1 levels (Fig. 7a). Survival analyses indicated that a higher expression of UBQLN1 was significantly associated with worse recurrence-free survival (RFS; *p* = 0.0461, hazard ratio [HR] = 0.5428). Moreover, patients with higher expressions of UBQLN1 were more likely to experience worse overall survival (OS), but with less significance (*p* = 0.1150, HR = 0.5381; Fig. 7b), likely due to the small sample size.

**Table 1:** Association between ubiquilin 1 expression and clinicopathological features

Variable	Ubiquilin 1 expression		*P*
	Low (%)	High (%)	
Age			>0.9999
<60 years	19 (51.4)	22 (53.7)	
≥60 years	18 (48.6)	19 (46.3)	
Sex			0.7480
Male	31 (83.8)	36 (87.8)	
Female	6 (16.2)	5 (12.2)	
HBsAg			0.7480
Negative	6 (16.2)	5 (12.2)	
Positive	31 (83.8)	36 (87.8)	
AFP (ng/ml)			0.8187
<400	23 (62.2)	24 (58.5)	
≥400	14 (37.8)	17 (41.5)	
Cirrhosis			>0.9999
No	16 (43.2)	17 (41.5)	
Yes	21 (56.8)	24 (58.5)	
Tumor size			0.8160
<5 cm	24 (64.9)	25 (61.0)	
≥5 cm	13 (35.1)	16 (39.0)	
Tumor number			0.1173
Single	37 (100)	37 (90.2)	
Multiple	0 (0)	4 (9.8)	
Tumor differentiation			0.5049
Well/moderately	16 (43.2)	21 (51.2)	
Poorly	21 (56.8)	20 (48.8)	
Tumor thrombi			0.5530
No	32 (86.5)	33 (80.5)	
Yes	5 (13.5)	8 (19.5)	
TNM stage			0.4357
I + II	35 (94.6)	36 (87.8)	
III + IV	2 (5.4)	5 (12.2)	
BCLC stage			0.2682
A	35 (94.6)	35 (85.4)	
B	2 (5.4)	6 (14.6)

To further validate the roles of UBQLN1 in vivo, sorafenib-resistant subcutaneous CDX models were established according to previous studies.^38^ Animal grade siRNA and lentivirus were used to suppress UBQLN1 expression (Fig. 7c). Consistent with the in vitro results, HepG2 and HCCLM3 xenografts with lower UBQLN1 levels were resensitized to sorafenib treatment compared with the control group (Fig. 7d–k).

Taken together, these clinical and in vivo data further demonstrate the role of UBQLN1 in promoting HCC tumor progression and sorafenib resistance.

## Discussion

Sorafenib and other emerging novel TKIs have been clinically effective for controlling HCC progression; however, drug resistance curtails their efficacy. Understanding the molecular mechanisms of sorafenib resistance may provide novel therapeutic targets and more insights for combination therapies for the management of HCC. Although multiple studies have demonstrated possible mechanisms underlying sorafenib resistance,^24,25,39,40^ the role of metabolic reprogramming and redox homeostasis in the process of sorafenib resistance has remained unknown.

Hypoxia is a common feature of solid tumors, such as HCC, that affects mitochondrial ETC complexes, resulting in increased ROS levels. ROS exhibit complicated effects on tumor development and progress. In the early stage during tumor initiation, ROS generally exerts pro-tumor effects, whereas in the late stage of tumor progression, excessive ROS production is cytotoxic for cancer cells because of the induction of metabolic stress.^41,42^ Sorafenib was reported to target ETC complexes that directly induce ROS generation in HCC cells.^18,19^ Therefore, to maintain sorafenib resistance and further facilitate tumor progression, cancer cells develop strategies to overcome excessive ROS production to obtain resistance to oxidative stress-induced cell death. In this study, we also confirmed that sorafenib treatment induced ROS generation in parental HCC cell lines. Unexpectedly, we found reduced ROS levels in sorafenib-resistant cells in response to sorafenib treatment, which contributes to the maintenance of resistance.

Mitochondria are the first site of ROS production and redox homeostasis, and hypoxia increases ROS levels mainly through affecting the mitochondria. Reduced mitochondrial biogenesis is likely to lead to reduced ROS production, thus a reduced stress for cells and a better adaption to sorafenib treatment. In this study, we found decreased mitochondrial biogenesis and ROS generation in sorafenib-resistant cells, indicating a critical role of reprogrammed ROS and redox metabolism in response to sorafenib treatment. Notably, our findings are consistent with a recent report from a genome-wide analysis of genes critical for sorafenib resistance.^43^ The study found that phosphoglycerate dehydrogenase (PHGDH) is a critical driver for HCC sorafenib resistance. Inactivation of PHGDH increases ROS levels and induces apoptosis in HCC cells upon sorafenib treatment. A lower mitochondrial volume is associated with the Warburg effect, which also contributes to tumor progression,^44^ as well as being a critical metabolic characteristic of cell stemness restoration,^45^ while both stemness maintenance and the Warburg effect contribute to sorafenib resistance.^46,47^ Another recent study^48^ suggested that mitochondrial TXNRD3 confers drug resistance via a redox-mediated mechanism, further supporting the findings of our current study. In addition, the alterations of biological redox couples vary in different cell lines, suggesting a cell line-specific, but generally reductive reprogramming in sorafenib-resistant cells and OE-PGC1β cells, compared with their controls.

Mitochondrial biogenesis is a complex process.^21^ Mitochondrial biogenesis is regulated by the transcriptional family of PGC1, which is composed of PGC1α, PGC1-related coactivator, and PGC1β. These proteins interact with other transcription factors involved in mitochondrial gene expression to regulate genes encoding proteins that are required for the transcription of the mitochondrial genome, such as TFAM. PGC1α and PGC1β share similar expression patterns and functions, and have key roles in regulating liver metabolism. They act as co-transcriptional factors to enhance mitochondrial biogenesis.^49^ However, their roles in cancer are not fully understood. Previous research revealed that PGC1α acts as a tumor suppressor in HCC,^50,51^ and it exerts its function mainly through mediating the Warburg effect by enhancing mitochondrial biogenesis.^44,52^ Other studies showed that PGC1α is regulated via the ubiquitin–proteasome pathway.^52,53^ However, the function of PGC1β has not been completely elucidated. PGC1β was previously reported to promote breast cancer tumor growth via SREBP1-mediated HKDC1 expression and induce apoptosis through mTOR-mediated signaling.^54,55^ In this study, increased ubiquitin-mediated proteolysis of PGC1β was found in sorafenib-resistant cells, which contributed to decreased mitochondrial biogenesis and ROS generation. We characterized a role for UBQLN1 in mediating PGC1β ubiquitination and degradation.

UBQLN1 has been widely studied in neurodegenerative diseases and was found to be deregulated in various disorders ranging from Alzheimer’s disease to cancer. One report demonstrated that UBQLN1 protects cells against ROS stress,^56^ but its role in cancer is unclear. Both downregulation and upregulation of UBQLN1 in cancer have been observed. As a UBL-UBA protein, UBQLN1 is assumed to bind to poly-ubiquitin chains of substrate and shuttle it to the proteasome. Some studies demonstrate that UBQLN1 facilitates the proteolysis of it bound substrates, while other studies also revealed that UBQLN1 stabilizes proteins that it binds, such as BCLb.^57^ These conflicting reports indicate that UBQLN1 either facilitates or retards the degradation of its bound substrates, which may account for the cell line-specific effect of UBQLN1 on general ubiquitination levels in HCC cell lines in the present study. In this study, we found that UBQLN1 serves as a degradation promoter through its chaperone function to facilitate the interaction between PGC1β and proteasome in a ubiquitination-independent manner. The target-dependent functions of UBQLN1 need further investigation.

In conclusion, this study revealed that UBQLN1-PGC1β-mediated mitochondrial biogenesis and ROS homeostasis play critical roles in sorafenib resistance in HCC, providing a potential signaling pathway and novel targets for combination therapies.

## Materials and methods

### Oxygen consumption rate detection

OCR measurements were performed using a Seahorse XF96 Extracellular Flux Analyzer to monitor mitochondrial respiration in real time, according to a previous report.^58^ Approximately 2 × 10^4^ cells were seeded into each well. For OCR measurements, after measuring the basal OCR, successive injections of 1 µM oligomycin, 0.5 µM FCCP, and 1 µM rotenone/5 µM antimycin were conducted to determine the respiratory capacity.

### Transmission electron microscopy

Cells were harvested after treatment and fixed with 2.5% glutaraldehyde at 4 °C overnight. The subsequent procedure followed a previous report.^59^ Briefly, after the first fixation, cells were washed with 0.1 M PBS three times followed by fixation in osmic acid at 4 °C for 1 h. Then, samples were subjected to dehydration in a graded ethanol series and embedded in acrylic resin. Sections were stained with uranyl acetate and lead citrate. Micrographs were acquired on a FEI Tecnai Spirit 120kv transmission electron microscope.

### Redox ratio measurement

NADH/NAD+ redox ratio was measured using NAD+/NADH Assay Kit with WST-8 (Beyotime, China). Cells were harvested and lysed according to instructions. Samples were separated into two parts. One part underwent 60 °C for 30 min to eliminate NAD+. Samples were treated with ethanol with ADH for 10 min at 37 °C. Incubation with working solution lasted for 30 min at 37 °C in dark condition. The absorption values were detected at a wavelength of 450 nm. NADH/NAD+ redox ratio and GSH/GSSG ratio were measured similarly using NADP+/NADPH Assay Kit, with WST-8 (Beyotime, China) and GSH and GSSG Assay Kit (Beyotime, China), according to the manufacturer’s protocol,

### Mass spectrum analysis

The peptide samples were analyzed on Thermo Fisher Exactive^TM^ plus Obitrap mass spectrometry. Mass spectrometry analysis were carried out at the Shanghai Bioprofile Technology Co., Ltd. (China) in the positive-ion mode with an automated data-dependent MS/MS analysis. The cellular component, molecular function, and biological process for the proteins were extracted and plotted with R. The KEGG pathways were analyzed by the database KEGG: Kyoto Encyclopedia of Genes and Genomes (http://www.kegg.jp/) and plotted with R.

### Sorafenib-resistant HCC mouse model

A subcutaneous mouse model was constructed, as previously described.^25^ In total, 100 million HCCLM3 cells were implanted into the flank of a BALB/c mouse. Then, the formed tumor was cut into small pieces of equal volume. These tissues were implanted into recipient 4-week-old BALB/c nude mice under anesthesia. Ten mice were used for this procedure. After 2 weeks, nine mice with similar tumor burdens were subjected to further experimentation, with five mice receiving 30 mg/kg/day sorafenib. After 8 weeks of treatment, mice were sacrificed, and tumor samples were collected for further investigation.

### HCC mouse model

Two subcutaneous cell-derived HCC mouse models were constructed using either HepG2 or HCCLM3 cells, as described previously.^24,25^ When tumors reached ~3 mm in diameter, the mice were randomly divided into two groups: (1) UBQLN1-downregulated group and (2) control group. UBQLN1 downregulation was conducted using siRNA or lentivirus transfection. siRNA transfection was performed using in vivo-grade cholesterol-conjugated RIG-I siRNA (RiboBio, China). Each tumor was locally injected with 5 nmol of siRNA twice a week for 2 weeks. Lentivirus transfection was performed by peritumoral injection of lentivirus overexpressing shRNA or control (10^7^ units in 50 μl PBS). All mice received 30 mg/kg/day sorafenib during the experiments. Two or 3 weeks after treatment, mice were sacrificed and subjected to further investigation.

The size of subcutaneous tumor was measured every week as follows: tumor volume (mm^3^) = (*L* × *W*^2^)/2, where *L* represents the long axis and *W* the short axis. All animal experiments were performed humanely in compliance with guidelines reviewed by the Animal Ethics Committee of the Biological Resource Centre of the Agency for Science, Technology, and Research at the Sir Run-Run Shaw Hospital, Zhejiang University School of Medicine.

### Clinical data analysis

Tissue samples were collected from 78 patients with complete follow-up information from the Department of General Surgery at Sir Run-Run Shaw Hospital. IHC was performed to evaluate UBQLN1 levels in tumor tissue. Patients were divided into low and high expression groups. The associations of UBQLN1 levels with the postsurgical prognoses of patients with HCC, as well as RFS and OS rates were analyzed using Kaplan–Meier analysis and the log-rank test.

### Statistical analysis

Statistical analysis was conducted using GraphPad Prism 8. Quantitative data between groups were compared using a *t* test or pair *t* test. Data were presented as the mean ± SEM from at least three independent experiments. RFS and OS curves were obtained using the Kaplan–Meier method, and differences were compared using the log-rank test. In all results, a two-tailed *p* value of <0.05 was considered statistically significant.

Additional materials and methods can be found in Supplementary Materials and Methods.

## Supplementary information

Supplementary Materials and Methods

## Data Availability

The additional data collected during this study are available from the corresponding author upon reasonable request.
