# Supplementary material for: UBQLN1 mediates sorafenib resistance through regulating mitochondrial biogenesis and ROS homeostasis by targeting PGC1β in hepatocellular carcinoma
Source: Signal Transduct Target Ther. 2021 May 18;6:190. doi: 10.1038/s41392-021-00594-4 (PMC8129126; doi:10.1038/s41392-021-00594-4)
Supplement: Supplementary file 1 — Supplementary Materials and Methods [file 41392_2021_594_MOESM1_ESM.docx]

Supplementary Materials for

Ubiquilin 1 Mediates Sorafenib Resistance through Regulating Mitochondrial Biogenesis and ROS Homeostasis by Targeting PGC1β in Hepatocellular Carcinoma

Authors: Junjie Xu^1-5,†^, Lin Ji^1-5,†^, Yeling Ruan^1-5,†^, Zhe Wan^1-5^, Zhongjie Lin^1-5^, Shunjie Xia^1-5^, Liye Tao^1-5^, Junhao Zheng^1-5^, Liuxin Cai^1-5^, Yifan Wang^1-5,^*, Xiao Liang^1-5,^*, and Xiujun Cai^1-5,^*

Correspondence to: Xiujun Cai (srrsh_cxj@zju.edu.cn), Xiao Liang (srrshlx@zju.edu.cn) and Yifan Wang(anwyf@zju.edu.cn).

†These authors contributed equally to this work.

**This PDF file includes:**

Supplementary Materials and Methods

Materials and Methods

Cell lines and cell culture

In this study, five human HCC cell lines (HepG2, HCC-LM3, SK-Hep-1, SNU-182, SNU-449) were purchased from the American Type Culture Collection (USA). Cell culture was performed according to the manufacturer’s protocol, and cells were grown in DMEM (Gibco, USA) or RPMI 1640 (Gibco, USA) supplemented with 10% FBS at 37°C in a 5% CO2 atmosphere. As most of the previous studies demonstrated, the *in vitro* establishment of sorafenib-resistant cell lines was accomplished by continuous administration of gradually increasing sorafenib concentration over 6 months as we previous reported. Thus, established resistant cell lines were defined as those could tolerate higher doses of sorafenib treatment compared with their parental counterparts. The resistant cells were maintained in the presence of sorafenib (5 μM for LM3-SR cells, 5 μM for SK-Hep-1-SR and HepG2-SR cells). The IC50 values for each cell lines were: 6.05 μM for LM3 cells, 11.86 μM for LM3-SR cells, 1.77 μM for SK-Hep-1 cells, 6.42 μM for SK-Hep-1-SR cells, 2.53 μM for HepG2 cells and 7.97 μM for HepG2-SR cells. Sorafenib, MG132 and cycloheximide (CHX) were purchased from MedChemExpress (USA). GSDetectTM Taqman Mycoplasma Detection Kit (UNG plus) were purchased from Geneseed (China).

Cell viability measurement

Cell growth inhibition was measured using a real-time cell analysis system (xCELLigence) according to the manufacturer’s instructions. Approximately 5000 cells/well were initially seeded. Cell growth data were collected every 5 min automatically.

Cell viability was also measured using the CCK-8 assay (Yeasen Biotech, China). Approximately 5000 cells/well were seeded in a 96-well plate. After the respective treatments, cells were incubated in working solution (10 µl of CCK-8 assay solution and 100 µl of DMEM) for approximately 1 h. The absorption values were detected at a wavelength of 450 nm. No-cell wells were used as blank controls.

Quantitative real-time PCR (qRT-PCR)

Total RNAs from tumor tissue or cells were extracted using TRIzol reagent (Invitrogen, USA) according to the manufacturer’s instructions. Complementary DNA (cDNA) was synthesized from approximately 1 µg of RNA from each sample using Hifair® Ⅱ 1st Strand cDNA Synthesis SuperMix for qPCR (gDNA digester plus) (Yeasen Biotech). Total DNAs from cells were extracted using an AxyPrep™ Multisource Genomic DNA Miniprep Kit (Axygen, USA). qRT-PCR was performed on a LightCycler® 480 real-time PCR system (Roche Applied Science, Germany) using Hieff UNICON® qPCR SYBR Green Master Mix (Yeasen Biotech). The relative mRNA expression or DNA content was calculated using the ΔΔCt method. Primers used in this study are listed in supplementary data.

Primer:

| Mitochondrial DNA content analysis: | |
| --- | --- |
| RC-21P-1 D-loop-F | CTAAATAGCCCACACGTTCCC |
| RC-21P-1 D-loop-R | AGAGCTCCCGTGAGTGGTTA |
| B2M-F | GCTGGGTAGCTCTAAACAATGTATTCA |
| B2M-R | CCATGTACTAACAAATGTCTAAAATGGT |
|  | |
| TFAM-F | GGTGCAGACAGGACAGTGTT |
| TFAM-R | CCCTACTCCCAGACTGCTCT |
| PGC1β-F | TCTGACGTGGACGAGCTTTC |
| PGC1β-R | CTTGCTGTTGGGGAGGATGT |
| ACTNB-F | TTAGTTGCGTTACACCCTTTC |
| ACTNB-R | ACCTTCACCGTTCCAGTT |

Western blotting (WB)

Total proteins were extracted from cells using RIPA lysis buffer (Beyotime, China) with protease inhibitor cocktail and phosphatase inhibitor cocktail (Medchemexpres, USA). Proteins were separated by sodium dodecyl sulfate-polyacrylamide gel electrophoresis and transferred to the PVDF membrane (Millipore, USA). Then, the membrane was blocked in skim milk (BD, USA). for 1 h at room temperature, followed by incubation with antibody overnight at 4°C. Membranes were rinsed with TBST the next day, incubated with secondary antibody for 1 h at room temperature. The antigen-antibody complex on the membrane was detected with enhanced chemiluminescence regents (Fdbio science, China).

Antibody and immunomagnetic beads:

| LC3B | CST | #12741 |
| --- | --- | --- |
| β-tubulin | FUDE Biological Technology | FD0064 |
| PGC1α | Abcam | Ab54481 |
| PGC1β | Abcam | Ab176328 |
| PGC1β | Abcam | Ab118603 |
| PARKIN | CST | #4211 |
| VDAC | Abcam | Ab154856 |
| PINK | CST | #6946 |
| Ubiquilin 1 | CST | #14526 |
| Ubiquilin 1 | Abcam | Ab128011 |
| Flag | Abcam | Ab205606 |
| Ubiquitin | SANTA CRUZ BIOTECHNOLOGY | Sc-8017 |
| HA | Sigma | H6908 |
| Anti-HA immunomagnetic beads | bimake.cn | B26201 |

Flow cytometry

Intracellular ROS levels were measured using a DCFH-DA probe (Sigma, USA) via flow cytometry. After respective treatment, cells were harvested and washed with PBS. Then cells were incubated with 10 µM DCFH-DA for 30 min at 37°C. in the dark condition. The measurement should be finished in a short time. The apoptosis ratio was measured using an Annexin V-FITC/PI apoptosis kit (Multi Science, China) according to the manufacturer’s protocol. Cells were incubated with 5 µl Annexin V-FITC and 10 µl PI for 5 min in the dark condition. Mitochondrial membrane potential (MMP) was measured using a Mitochondria Staining Kit (JC-1) (Multi Science). Cells were incubated with 2 µM JC-1 for 15 min at 37°C in a 5% CO2 atmosphere without lighting. All flow cytometry analyses were performed using a BD LSRFortessa cell analyzer (BD Biosciences, USA). Analysis was conducted FlowJo software.

Fluorescence microscope

To further demonstrate autophagic flux, cells were transfected with GFP-RFP-LC3 double-labeled adenovirus (Vigene Bioscience, China). This tandem RFP-GFP sensor capitalizes on the pH difference between the acidic autolysosome and the neutral autophagosome and the pH sensitivity differences exhibited by GFP (green fluorescent protein) and RFP (red fluorescent protein) to monitor progression from the autophagosome to autolysosome (autophagic flux). By combining an acid-sensitive GFP with an acid-insensitive RFP, the change from autophagosome (neutral pH) to autolysosome (with an acidic pH) can be visualized by imaging the specific loss of the GFP fluorescence, leaving only red fluorescence. Then, cells were seeded into 6-well plates. After respective treatments, fluorescent images were obtained using a Zeiss fluorescence microscope and ZEN software.

Confocal microscopy was used to localize UBQLN1 and PGC1β. Immunofluorescent co-localization was performed as previous report ^25^. Briefly, cells were fixed with 4% paraformaldehyde for 20 min, rinsed with PBS, permeabilized with 0.2% triton X-100 in PBS for 10 min, blocked with 10% goat serum and incubated with anti-UBQLN1 antibody (Abcam, UK) diluted 1:250 in blocking solution for 1h under room temperature. After being washed with PBS for three times, cells were incubated with Goat anti-Rabbit IgG secondary antibody conjugated with Dylight649 (Multi sciences, China) for 1 h under room temperature. After being washed with PBS, cells were then incubated with anti-DDDDK tag antibody (Abcam, UK) for 1 h under room temperature, rinsed with PBS, incubated with Goat anti-Mouse IgG secondary antibody conjugated with Alexa Fluor 546 for 1h. After being washed with PBS, cells were then incubated with DAPI (Beyotime, China) for 4min. Images were collected with a Nikon A1 camera (Nikon, Japan).

Co-immunoprecipitation

Co-immunoprecipitation was performed in HepG2-SR according to previous reports. Cells were lysed with western and IP buffer (Beyotime, China) with Protease Inhibitor Cocktail (Medchemexpress, USA). Anti-HA immunomagnetic beads or Protein A/G Agarose Beads (Santa Cruz, USA) coated with 5 mg of normal antibodies against rabbit immunoglobulin G (Beyotime, China) and UBQLN1 (CST, USA) were incubated with cell lysates overnight at 4°C. Beads were collected and washed for several times and then subjected to protein extraction using RIPA.

Immunohistochemistry (IHC)

Paraffin-embedded specimens were cut into 4-µm slides. IHC was performed as described previously. After rehydration, antigen retrieval was conducted by boiling samples in 0.01 M citrate buffer for 5 min. Hydrogen peroxide was used to block peroxidase, followed by 10% goat serum incubation. The primary antibody against Ubiquilin 1 was diluted to 1:500. After overnight incubation at 4°C in a humidified chamber, IHC staining and DAB visualization for tissues were detected using the GTVision III detection system (Gene Tech, China). The staining intensity was graded using the German immunoreactive score [23]. Staining intensity was graded as “0” (negative), “1” (weak), “2” (moderate), or “3” (strong) by two pathologists without prior knowledge of the clinical information. Scores 0 or 1 were classified as low expression, and those of 2 or 3 were classified as high expression.

Gene over-expression

CDS sequence of PGC1β was composed by TSINGKE Biological Technology (China). CDS sequence of UBQLN1 was cloned from cDNA using Phanta® Master Mix (Vazyme, China). pXF4H was kindly provided by Prof. Feng Xinhua from Zhejiang university, school of Medicine. Sequence of myc-ubquitin was cloned into pXF4H between ClaI and EcoR1. Sequences of Myc-ubquitin and pXF4H were provided below. Sequences were cloned into PCDH plasmid with ClonExpress II One Step Cloning Kit (Vazyme, China). Lentivirus was constructed using the transfer plasmid PCDH and the packaging plasmids pMD2.G (Addgene, UK) and psPAX2 (Addgene, UK). Supernatant containing lentivirus was collected for 48 h. Lentiviral infection of the cell lines were carried through for 24 h. After another 24 h, 1 mg/ml puromycin was added to the medium for selection.

Oligonucleotide transfection

siRNA targeting Ubiquilin and normal control siRNA were obtained from Ribobio (Guangzhou, China). siRNAs were transiently transfected into HCC cells at a working concentration of 50 nM using Lipofectamine 3000 reagent (Invitrogen, USA) according to the manufacturer’s protocol. The gene-silencing effect was confirmed by qPCR and WT at 48–72 h after transfection. The sequences of siRNAs are listed in the Supplementary data.

siRNA sequence:

| siUBQLN1 | AACUGAAAUCUGACUUCUGGAUU |
| --- | --- |
|  | UCCAGAAGUCAGAUUUCAGUUUU |
| siNC | UUCUCCGAACGUGUCACGUTT |
|  | ACGUGACACGUUCGGAGAATT |

Relevant plasmid and sequence information:

pXF4H:

ATC GAT(CalI)……GAA TTC(EcoRI) GAT ATC AGA TCT GCG GCC GCA GCT AGC CTC GAG GAT CCT CTA GAG TCG ACC TGC AGA AGC TT

pXF6F:

(ATG-3xFlag) GAA TTC (EcoRI) GAT ATC AGA TCT GCG GCC GCA GCT AGC CTC GAG GAT CC(BamHI)T CTA GAG TCG ACC TGC AGA AGC TT

3XFlag:

GACTACAAAGACCATGACGGTGATTATAAAGATCATGACATCGACTACAAGGATGACGATGACAAG

3XHA：

TACCCTTACGACGTCCCAGACTACGCTGGATCCTACCCTTACGACGTCCCAGACTACGCTTACCCTTACGACGTCCCAGACTACGCT

Ubiquitin CDS：

ATGCAGATCTTCGTCAAGACGTTAACCGGTAAAACCATAACTCTAGAAGTTGAATCTTCCGATACCATCGACAACGTTAAGTCGAAAATTCAAGACAAGGAAGGCATTCCACCTGATCAACAAAGATTGATCTTTGCCGGTAAGCAGCTCGAGGACGGTAGAACGCTGTCTGATTACAACATTCAGAAGGAGTCGACCTTACATCTTGTCTTAAGACTAAGAGGTGGTTGA

UBQLN1 CDS：

ATGGCCGAGAGTGGTGAAAGCGGCGGTCCTCCGGGCTCCCAGGATAGCGCCGCCGGAGCCGAAGGTGCTGGCGCCCCCGCGGCCGCTGCCTCCGCGGAGCCCAAAATCATGAAAGTCACCGTGAAGACCCCGAAGGAAAAGGAGGAATTCGCCGTGCCCGAGAATAGCTCCGTCCAGCAGTTTAAGGAAGAAATCTCTAAACGTTTTAAATCACATACTGACCAACTTGTGTTGATATTTGCTGGAAAAATTTTGAAAGATCAAGATACCTTGAGTCAGCATGGAATTCATGATGGACTTACTGTTCACCTTGTCATTAAAACACAAAACAGGCCTCAGGATCATTCAGCTCAGCAAACAAATACAGCTGGAAGCAATGTTACTACATCATCAACTCCTAATAGTAACTCTACATCTGGTTCTGCTACTAGCAACCCTTTTGGTTTAGGTGGCCTTGGGGGACTTGCAGGTCTGAGTAGCTTGGGTTTGAATACTACCAACTTCTCTGAACTACAGAGTCAGATGCAGCGACAACTTTTGTCTAACCCTGAAATGATGGTCCAGATCATGGAAAATCCCTTTGTTCAGAGCATGCTCTCAAATCCTGACCTGATGAGACAGTTAATTATGGCCAATCCACAAATGCAGCAGTTGATACAGAGAAATCCAGAAATTAGTCATATGTTGAATAATCCAGATATAATGAGACAAACGTTGGAACTTGCCAGGAATCCAGCAATGATGCAGGAGATGATGAGGAACCAGGACCGAGCTTTGAGCAACCTAGAAAGCATCCCAGGGGGATATAATGCTTTAAGGCGCATGTACACAGATATTCAGGAACCAATGCTGAGTGCTGCACAAGAGCAGTTTGGTGGTAATCCATTTGCTTCCTTGGTGAGCAATACATCCTCTGGTGAAGGTAGTCAACCTTCCCGTACAGAAAATAGAGATCCACTACCCAATCCATGGGCTCCACAGACTTCCCAGAGTTCATCAGCTTCCAGCGGCACTGCCAGCACTGTGGGTGGCACTACTGGTAGTACTGCCAGTGGCACTTCTGGGCAGAGTACTACTGCGCCAAATTTGGTGCCTGGAGTAGGAGCTAGTATGTTCAACACACCAGGAATGCAGAGCTTGTTGCAACAAATAACTGAAAACCCACAACTGATGCAAAACATGTTGTCTGCCCCCTACATGAGAAGCATGATGCAGTCACTAAGCCAGAATCCTGACCTTGCTGCACAGATGATGCTGAATAATCCCCTATTTGCTGGAAATCCTCAGCTTCAAGAACAAATGAGACAACAGCTCCCAACTTTCCTCCAACAAATGCAGAATCCTGATACACTATCAGCAATGTCAAACCCTAGAGCAATGCAGGCCTTGTTACAGATTCAGCAGGGTTTACAGACATTAGCAACGGAAGCCCCGGGCCTCATCCCAGGGTTTACTCCTGGCTTGGGGGCATTAGGAAGCACTGGAGGCTCTTCGGGAACTAATGGATCTAACGCCACACCTAGTGAAAACACAAGTCCCACAGCAGGAACCACTGAACCTGGACATCAGCAGTTTATTCAGCAGATGCTGCAGGCTCTTGCTGGAGTAAATCCTCAGCTACAGAATCCAGAAGTCAGATTTCAGCAACAACTGGAACAACTCAGTGCAATGGGATTTTTGAACCGTGAAGCAAACTTGCAAGCTCTAATAGCAACAGGAGGTGATATCAATGCAGCTATTGAAAGGTTACTGGGCTCCCAGCCATCATAG

PGC1β CDS：

ATGGCGGGGAACGACTGCGGCGCGCTGCTGGACGAAGAGCTCTCCTCCTTCTTCCTCAACTATCTCGCTGACACGCAGGGTGGAGGGTCCGGGGAGGAGCAACTCTATGCTGACTTTCCAGAACTTGACCTCTCCCAGCTGGATGCCAGCGACTTTGACTCGGCCACCTGCTTTGGGGAGCTGCAGTGGTGCCCAGAGAACTCAGAGACTGAACCCAACCAGTACAGCCCCGATGACTCCGAGCTCTTCCAGATTGACAGTGAGAATGAGGCCCTCCTGGCAGAGCTCACCAAGACCCTGGATGACATCCCTGAAGATGACGTGGGTCTGGCTGCCTTCCCAGCCCTGGATGGTGGAGACGCTCTATCATGCACCTCAGCTTCGCCTGCCCCCTCATCTGCACCCCCCAGCCCTGCCCCGGAGAAGCCCTCGGCCCCAGCCCCTGAGGTGGACGAGCTCTCACTGCTGCAGAAGCTCCTCCTGGCCACATCCTACCCAACATCAAGCTCTGACACCCAGAAGGAAGGGACCGCCTGGCGCCAGGCAGGCCTCAGATCTAAAAGTCAACGGCCTTGTGTTAAGGCGGACAGCACCCAAGACAAGAAGGCTCCCATGATGCAGTCTCAGAGCCGAAGTTGTACAGAACTACATAAGCACCTCACCTCGGCACAGTGCTGCCTGCAGGATCGGGGTCTGCAGCCACCATGCCTCCAGAGTCCCCGGCTCCCTGCCAAGGAGGACAAGGAGCCGGGTGAGGACTGCCCGAGCCCCCAGCCAGCTCCAGCCTCTCCCCGGGACTCCCTAGCTCTGGGCAGGGCAGACCCCGGTGCCCCGGTTTCCCAGGAAGACATGCAGGCGATGGTGCAACTCATACGCTACATGCACACCTACTGCCTCCCCCAGAGGAAGCTGCCCCCACAGACCCCTGAGCCACTCCCCAAGGCCTGCAGCAACCCCTCCCAGCAGGTCAGATCCCGGCCCTGGTCCCGGCACCACTCCAAAGCCTCCTGGGCTGAGTTCTCCATTCTGAGGGAACTTCTGGCTCAAGACGTGCTCTGTGATGTCAGCAAACCCTACCGTCTGGCCACGCCTGTTTATGCCTCCCTCACACCTCGGTCAAGGCCCAGGCCCCCCAAAGACAGTCAGGCCTCCCCTGGTCGCCCGTCCTCGGTGGAGGAGGTAAGGATCGCAGCTTCACCCAAGAGCACCGGGCCCAGACCAAGCCTGCGCCCACTGCGGCTGGAGGTGAAAAGGGAGGTCCGCCGGCCTGCCAGACTGCAGCAGCAGGAGGAGGAAGACGAGGAAGAAGAGGAGGAGGAAGAGGAAGAAGAAAAAGAGGAGGAGGAGGAGTGGGGCAGGAAAAGGCCAGGCCGAGGCCTGCCATGGACGAAGCTGGGGAGGAAGCTGGAGAGCTCTGTGTGCCCCGTGCGGCGTTCTCGGAGACTGAACCCTGAGCTGGGCCCCTGGCTGACATTTGCAGATGAGCCGCTGGTCCCCTCGGAGCCCCAAGGTGCTCTGCCCTCACTGTGCCTGGCTCCCAAGGCCTACGACGTAGAGCGGGAGCTGGGCAGCCCCACGGACGAGGACAGTGGCCAAGACCAGCAGCTCCTACGGGGACCCCAGATCCCTGCCCTGGAGAGCCCCTGTGAGAGTGGGTGTGGGGACATGGATGAGGACCCCAGCTGCCCGCAGCTCCCTCCCAGAGACTCTCCCAGGTGCCTCATGCTGGCCTTGTCACAAAGCGACCCAACTTTTGGCAAGAAGAGCTTTGAGCAGACCTTGACAGTGGAGCTCTGTGGCACAGCAGGACTCACCCCACCCACCACACCACCGTACAAGCCCACAGAGGAGGATCCCTTCAAACCAGACATCAAGCATAGTCTAGGCAAAGAAATAGCTCTCAGCCTCCCCTCCCCTGAGGGCCTCTCACTCAAGGCCACCCCAGGGGCTGCCCACAAGCTGCCAAAGAAGCACCCAGAGCGAAGTGAGCTCCTGTCCCACCTGCGACATGCCACAGCCCAGCCAGCCTCCCAGGCTGGCCAGAAGCGTCCCTTCTCCTGTTCCTTTGGAGACCATGACTACTGCCAGGTGCTCCGACCAGAAGGCGTCCTGCAAAGGAAGGTGCTGAGGTCCTGGGAGCCGTCTGGGGTTCACCTTGAGGACTGGCCCCAGCAGGGTGCCCCTTGGGCTGAGGCACAGGCCCCTGGCAGGGAGGAAGACAGAAGCTGTGATGCTGGCGCCCCACCCAAGGACAGCACGCTGCTGAGAGACCATGAGATCCGTGCCAGCCTCACCAAACACTTTGGGCTGCTGGAGACCGCCCTGGAGGAGGAAGACCTGGCCTCCTGCAAGAGCCCTGAGTATGACACTGTCTTTGAAGACAGCAGCAGCAGCAGCGGCGAGAGCAGCTTCCTCCCAGAGGAGGAAGAGGAAGAAGGGGAGGAGGAGGAGGAGGACGATGAAGAAGAGGACTCAGGGGTCAGCCCCACTTGCTCTGACCACTGCCCCTACCAGAGCCCACCAAGCAAGGCCAACCGGCAGCTCTGTTCCCGCAGCCGCTCAAGCTCTGGCTCTTCACCCTGCCACTCCTGGTCACCAGCCACTCGAAGGAACTTCAGATGTGAGAGCAGAGGGCCGTGTTCAGACAGAACGCCAAGCATCCGGCACGCCAGGAAGCGGCGGGAAAAGGCCATTGGGGAAGGCCGCGTGGTGTACATTCAAAATCTCTCCAGCGACATGAGCTCCCGAGAGCTGAAGAGGCGCTTTGAAGTGTTTGGTGAGATTGAGGAGTGCGAGGTGCTGACAAGAAATAGGAGAGGCGAGAAGTACGGCTTCATCACCTACCGGTGTTCTGAGCACGCGGCCCTCTCTTTGACAAAGGGCGCTGCCCTGAGGAAGCGCAACGAGCCCTCCTTCCAGCTGAGCTACGGAGGGCTCCGGCACTTCTGCTGGCCCAGATACACTGACTACGATTCCAATTCAGAAGAGGCCCTTCCTGCGTCAGGGAAAAGCAAGTATGAAGCCATGGATTTTGACAGCTTACTGAAAGAGGCCCAGCAGAGCCTGCATTAG
